# Dual RNA-seq of *Xanthomonas oryzae* pv. *oryzicola* infecting rice reveals novel insights into bacterial-plant interaction

**DOI:** 10.1371/journal.pone.0215039

**Published:** 2019-04-17

**Authors:** Zhou-Xiang Liao, Zhe Ni, Xin-Li Wei, Long Chen, Jian-Yuan Li, Yan-Hua Yu, Wei Jiang, Bo-Le Jiang, Yong-Qiang He, Sheng Huang

**Affiliations:** State Key Laboratory for Conservation and Utilization of Subtropical Agro-bioresources, College of Life Science and Technology, Guangxi University, Nanning Guangxi, China; Shanghai Jiao Tong University, CHINA

## Abstract

The Gram-negative bacterium *Xanthomonas oryzae* pv. *oryzicola* (*Xoc*) is the causal agent of rice bacterial leaf streak (BLS), one of the most destructive diseases of rice (*Oryza sativa* L.) that is the important staple crop. *Xoc* can invade host leaves via stomata and wounds and its type three secretion system (T3SS) is pivotal to its pathogenic lifestyle. In this study, using a novel dual RNA-seq approach, we examined transcriptomes of rice and *Xoc* in samples inoculated with wild type *Xoc* GX01 and its T3SS defective strain (T3SD), to investigate the global transcriptional changes in both organisms. Compared with T3SD strain, rice inoculated with wild type *Xoc* GX01 resulted in significant expression changes of a series of plant defence related genes, including ones altered in plant signalling pathway, and downregulated in phenylalanine metabolism, flavonoid and momilactone biosynthesis, suggesting repression of plant defence response and reduction in both callose deposition and phytoalexin accumulation. Also, some known transcription activator-like effector (TALE) targets were induced by *Xoc* GX01, e.g. OsSultr3;6 which contributes to rice susceptibility. Some cell elongation related genes, including several expansin genes, were induced by GX01 too, suggesting that *Xoc* may exploit this pathway to weaken cell wall strength, beneficial for bacterial infection. On the other hand, compared with wild type, the T3SD strain transcriptome *in planta* was characterized by downregulation of ATP, protein and polysaccharide synthesis, and upregulation of antioxidation and detoxification related genes, revealing that T3SD strain faced serious starvation and oxidation stresses *in planta* without a functional T3SS. In addition, comparative global transcript profiles of *Xoc in planta* and in medium revealed an upregulation of virulence factor synthesis and secretion *in planta* in favour of bacterial infection. Collectively, this study provides a comprehensive representation of cross talk between the host and bacterial pathogen, revealing insights into the *Xoc*-rice pathogenic dynamic and reveals novel strategies exploited by this important pathogen to cause disease.

## Introduction

*Oryza sativa* L. (rice) is one of the world’s most important food crops and is cultivated in both tropical and temperate regions [1]. The Gram-negative bacterium *Xanthomonas oryzae* pv. *oryzicola* (*Xoc*) is the causative agent of bacterial leaf streak (BLS), one of the most destructive diseases of rice, which has contributed to significant yield losses (up to 30%) over the last decade [2–4]. Successful colonization by *Xoc* depends on its ability to adhere and adapt to the plant tissues, which serve as a frontline defence against infection. The pathogen enters rice leaves through stomata or wound sites and colonizes intercellular spaces in the mesophyll, resulting in water-soaked interveinal lesions that develop into translucent streaks[2–4]. *Xoc* does not invade the xylem, which is in contrast to other rice bacterial pathogens that cause bacterial blight by invading vascular tissues [2–4].

It is becoming more appreciated that bacterial plant pathogens encounter changes in the environmental conditions within different anatomical sites of the host, making rapid adaptation a crucial factor for survival and disease [5,6]. The course of infection triggers a dynamic cascade of events that culminates in alterations in gene expression patterns in both interacting bacterium and the plant [5,6]. Although several studies have focused on understanding the molecular mechanisms of adaptation in various bacterial plant pathogens and/or their hosts, the interaction between *Xoc* and rice (plant host) during infection remains poorly detailed.

Several functional genomic studies have demonstrated that the type three-secretion system (T3SS) plays a key role in *Xoc* infection of rice [7–9]. It is now well known that the T3SS is a complex transmembrane structure that can secrete proteins called type three secreted effectors (T3SEs) that can manipulate host cell physiology [9]. Specifically, these T3SEs can mediate effector-triggered susceptibility (ETS) or effector triggered immunity (ETI) that usually results in host gene expression changes [9]. Consequently, transcriptional reprogramming of plant cells is considered to be central to plant defense [9]. Therefore, understanding this complex interplay is a main aim of most current plant-pathogen interaction studies as it is felt that such insights will support the development of new approaches for disease control and plant health.

Several studies of bacterial human infection have taken advantage of massively parallel cDNA sequencing (dual RNA-seq) as it offers a comprehensive and simultaneous whole-genome transcriptional profile of both the host and the invading pathogen and overcomes the existing technical and economical limitations of probe-dependent methods [10]. Despite these technological advances in studying human infection there have only been sparingly applied to plant disease due to lack of robust sampling models [11]. Using this improved technology, we developed a rice plant infection model to monitor the progression of *Xoc* infection of rice and simultaneously analyse the global gene expression of bacteria and host by dual RNA sequencing.

## Results and discussion

### Dual RNA-Seq of Plant-Bacteria mixed samples

**Fig 1:**
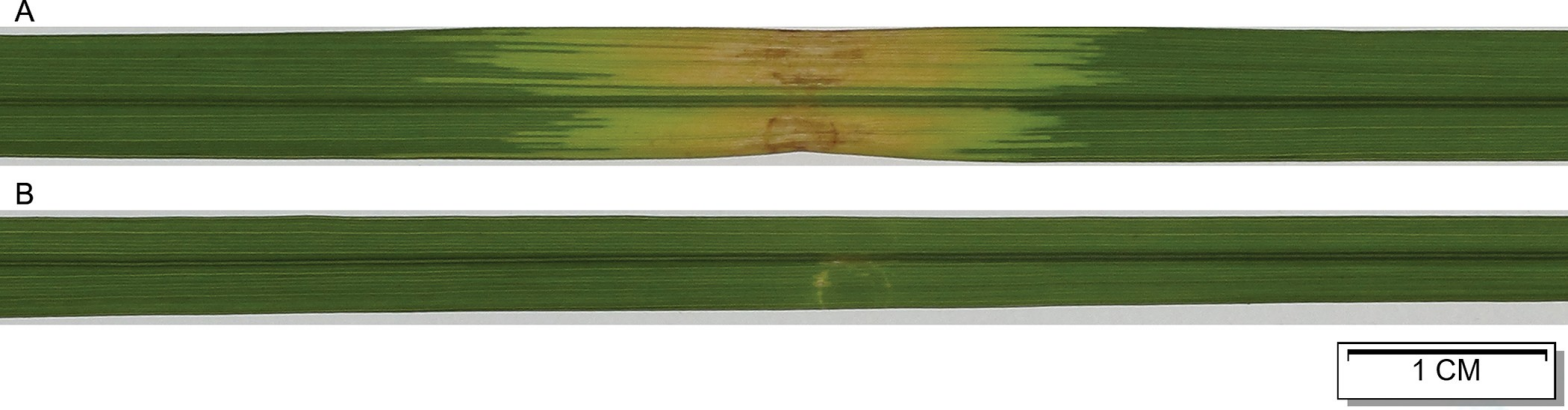
Rice leaf lesions caused by *Xoc* GX01 and its T3SS-defective mutant. 14 days after inoculated, the wild type *Xoc* GX01 (A) caused serious lesions on rice leave but its T3SS-defective mutant didn’t (B).

The susceptible rice *Oryza sativa* L. cv. Nipponbare was specifically chosen to be inoculated with a virulent *Xoc* strain from Hezhou, Guangxi, China (designated *Xoc* GX01) and a T3SS-defective mutant (designated *Xoc* GX01 T3SD). As the T3SS defective mutant cannot deliver any T3SE into rice, *Xoc* GX01 T3SD did not cause any visible lesion on rice leaf after inoculation (Fig 1). Therefore, comparing the gene express profile between these two kinds of rice samples can reveal the impacts done by T3SEs in *Xoc* to rice gene expressions and what kind of changes should benefit *Xoc* at the same time.

**Fig 2:**
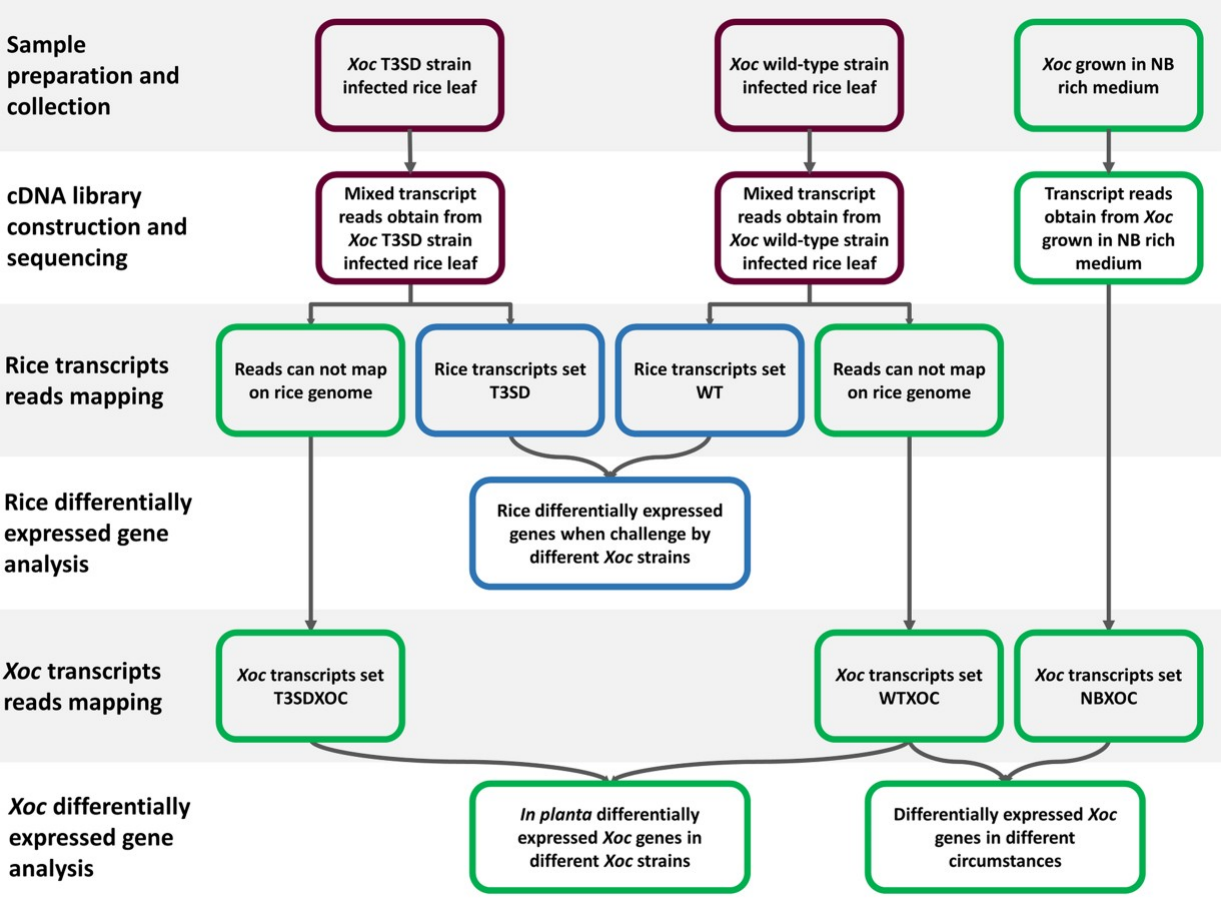
Schematic description of study design and mapping approach.

Our dual RNA-seq method was used to obtain *in situ* real-time transcriptome data from mixed samples recovered from infiltration-inoculated rice leaves (See Methods). To ensure coverage of the *Xoc* transcriptome and overcome the RNA quantity differences between the rice and infecting bacterium, we carried out sequencing to a depth of >20 GB per mixed sample (Table 1). After library sequencing, a “host first, pathogen following” mapping strategy was used to chart the obtained sequence reads onto the two species (Fig 2). The reads obtained were mapped first onto the reference genome of *Oryza sativa* L. cv. Nipponbare. We obtained more than 170 million reads that mapped onto the rice genome (Table 2). Subsequently, the unmapped reads obtained from the mixed sample cDNA libraries were charted onto *Xoc* BLS256 reference genome. During this process, more than 2 million reads from wild-type-infected rice leaf samples were mapped onto the *Xoc* genome (Table 3), which was sufficient for the DEG (differentially expressed genes) analysis. However, after infection with the T3SS-defective mutant, only about half a million reads were obtained from the rice leaf samples could be mapped onto the *Xoc* BLS256 genome, which was not ideal but still viable for subsequent analysis (Table 3). In parallel, to our *in planta* studies we collected wild-type *Xoc* transcriptome data from *Xoc* GX01 and *Xoc* GX01 T3SD grown in rich medium (Tables 1 and 3) as this would provide a data set to contrast expression changes seen in the *in planta* environment.

**Table 1:** RNA-seq reads statistics.

Identifier	Raw reads	Clean reads	Error rate (%)	Q20 (%)	Q30 (%)	GC content (%)
WT1_1	95,610,695	93,512,704	0.03	97.55	94.93	45.31
WT1_2	95,610,695	93,512,704	0.03	95.82	92	45.12
WT2_1	94,235,807	91,839,851	0.03	97.56	94.95	45.29
WT2_2	94,235,807	91,839,851	0.03	96.05	92.41	45.13
T3SD1_1	89,734,396	87,172,389	0.03	97.48	94.8	45.25
T3SD1_2	89,734,396	87,172,389	0.03	95.84	92.02	45.04
T3SD2_1	94,275,426	91,687,426	0.03	97.55	94.92	44.96
T3SD2_2	94,275,426	91,687,426	0.03	95.96	92.24	44.79
NBXOC1	5,415,171	5,258,414	0.03	97.02	93.42	62.79
NBXOC2	5,415,171	5,258,414	0.04	94.55	89.47	61.94

Note: WT1_1 and WT1_2 represent one biological replicate of wild type *Xoc* GX01 infected leave sample WT1 sequenced by two different primers. Same as other identifiers except NBXOC1 and NBXOC2, which represent one biological replicate of wild type *Xoc* GX01 in rich medium culture NBXOC sequenced by two different primer.

**Table 2:** RNA-seq mapping statistics of mixed sample reads mapped on Rice (*Oryza sativa* L. cv. Nipponbare).

Identifier	Total reads	Total mapped	Multiple mapped	Uniquely mapped	Reads map to '+'	Reads map to '-'	Non-splice reads	Splice reads
WT1	187,025,408	176,421,721 (94.33%)	44,490,526 (23.79%)	131,931,195 (70.54%)	65,777,472 (35.17%)	66,153,723 (35.37%)	115,051,498 (61.52%)	16,879,697 (9.03%)
WT2	183,679,702	172,110,901 (93.70%)	41,781,368 (22.75%)	130,329,533 (70.95%)	65,003,461 (35.39%)	65,326,072 (35.57%)	113,149,978 (61.60%)	17,179,555 (9.35%)
T3SD1	174,344,778	161,994,505 (92.92%)	40,639,300 (23.31%)	121,355,205 (69.61%)	60,530,735 (34.72%)	60,824,470 (34.89%)	104,223,315 (59.78%)	17,131,890 (9.83%)
T3SD2	183,374,852	174,067,107 (94.92%)	41,790,977 (22.79%)	132,276,130 (72.13%)	65,976,701 (35.98%)	66,299,429 (36.16%)	115,835,445 (63.17%)	16,440,685 (8.97%)

**Table 3:** RNA-seq mapping statistics of reads mapped on *Xoc* (*Xanthomonas oryzae* pv. *oryzicola* BLS256).

Identifier	Total reads	Total mapped	Multiple mapped	Uniquely mapped	Reads map to '+'	Reads map to '-'
WT1XOC	9,607,188	2,176,115 (22.65%)	158,226 (1.65%)	2,017,889 (21.00%)	1,008,875 (10.50%)	1,009,014 (10.50%)
WT2XOC	10,376,968	2,254,720 (21.73%)	152,662 (1.47%)	2,102,058 (20.26%)	1,050,911 (10.13%)	1,051,147 (10.13%)
T3SD1XOC	10,525,642	461,053 (4.38%)	35,700 (0.34%)	425,353 (4.04%)	212,678 (2.02%)	212,675 (2.02%)
T3SD2XOC	8,084,774	537,825 (6.65%)	42,713 (0.53%)	495,112 (6.12%)	247,493 (3.06%)	247,619 (3.06%)
NBXOC	10,328,618	10,076,374 (97.56%)	514,736 (4.98%)	9,561,638 (92.57%)	4,778,561 (46.27%)	4,783,077 (46.31%)

**Fig 3:**
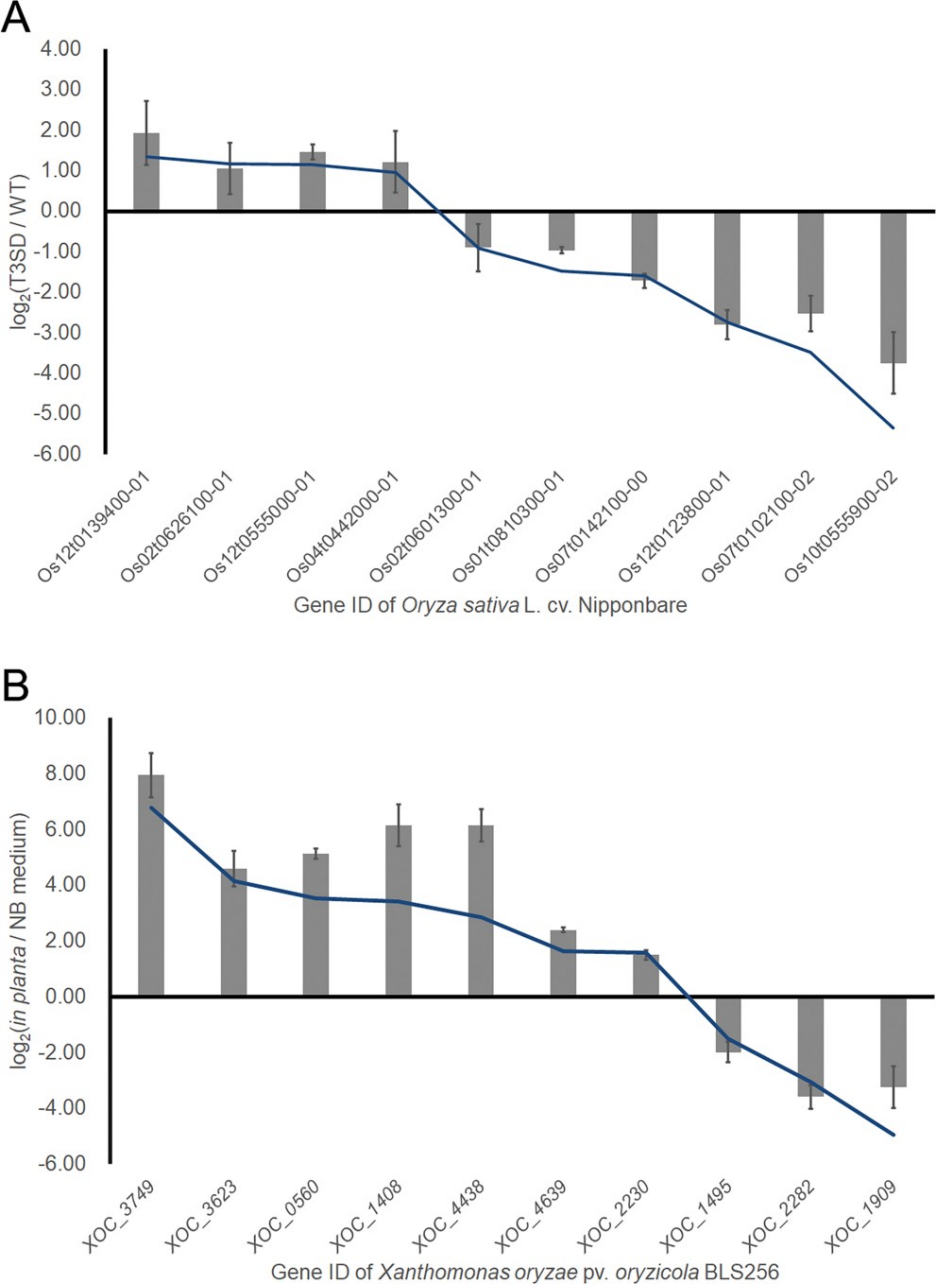
Verification of DEGs using qRT-PCR. (A)The line shows the differences in the expression levels resulting from RNA-seq in rice leaves infected with the GX01 T3SD and GX01 wild-type (WT) strains represented as log2(FPKMT3SD/FPKMWT). The columns show expression level differences based on qRT-PCR assay results (three biological replicates). (B) The line shows the differences in the expression levels resulting from RNA-seq and displays the fold changes of GX01 growing in planta compared to in media represented as log2(FPKM *in planta*/FPKM *in media*). The columns show expression level differences in the qRT-PCR assay results (three biological replicates). All qPCR data were processed using the 2^(-ΔΔC(t))^ method.

To quantify the gene expression levels, the fragments per kilobase of transcript per million reads mapped (FPKM) of each gene were calculated. We grouped the gene expression data from the Rice Differential Group (RDG, rice infected with wild-type *Xoc* GX01 and *Xoc* GX01 T3SD) and the *Xanthomonas* Differential Group (XDG, *Xoc* GX01 grown in different environments) for the differential gene expression analysis. Then, to verify that our dual RNA-seq data were accurate, a series of qRT-PCR assays were used to confirm the gene expression changes revealed. By randomly picking and quantifying 10 genes from the RDG and XDG groups, we demonstrated that the dual RNA-seq data were robust (Fig 3, S1 Table).

### Analysis of differential expression genes within mixed samples

**Fig 4:**
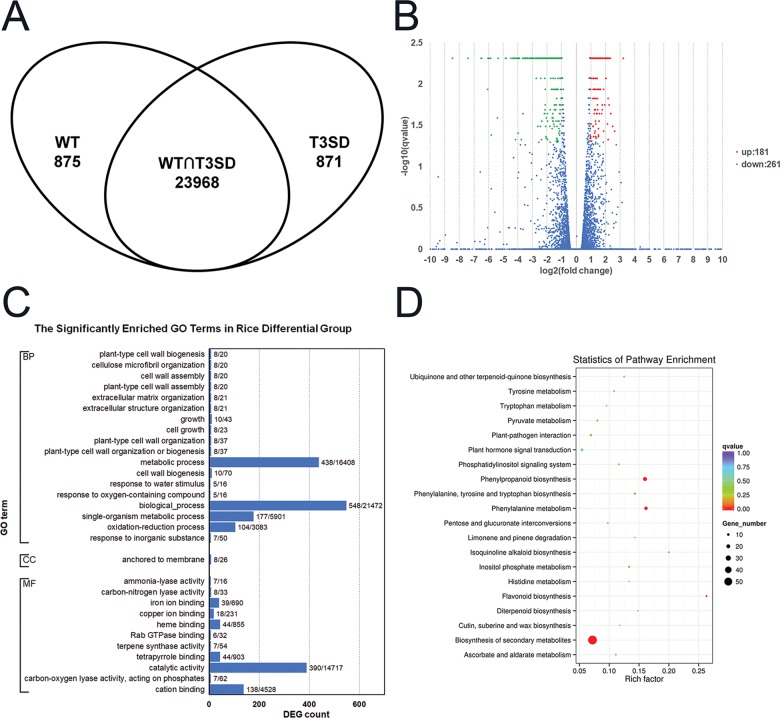
Differential gene expression (DEG) of *Oryza sativa* L. cv. Nipponbare infected by wild-type *Xanthomonas oryzae* pv. *Oryzicola* and a T3SS-defective mutant. **(A)** The Venn diagram shows DEGs between rice leaves infected with the GX01 wild-type (WT) and GX01 T3SD strains. The data represents the numbers of genes with FPKM > 1. **(B)** The volcano plot shows DEGs between the GX01 wild-type (WT) and T3SD strains. The WT served as the control. A log2(fold change) > 1 and q-value < 0.05 were used as cutoff values. **(C)** Histogram showing GO enrichment analysis of DEGs in the Rice Differential Group (RDG). The y-axis indicates the GO terms, and the x-axis indicates the numbers of DEGs shown as the DEG number/background number. All terms shown have corrected P-values < 0.05. BP: biological process; CC: cellular component; MF: molecular function. **(D)** Bubble chart showing KEGG enrichment analysis of DEGs in the Rice Differential Group (RDG). The y-axis indicates the enriched pathways, and the x-axis indicates the rich factor. Bubble color indicates q-value and the size indicates the DEG count. The chart showed most enriched 20 pathways.

After gene expression quantification, we confirmed there are 27620 rice (infected with wild-type *Xoc* GX01 and *Xoc* GX01 T3SD) transcripts in our libraries, including 25714 annotated genes, 980 novel genes and 1086 long non-coding RNAs. We excluded novel genes and long non-coding RNAs and only use transcripts which mapped onto annotated genes for the following gene expression analysis. While 23968 gene transcripts were present in both samples, 875 were unique to the wild-type *Xoc* GX01 infiltrated rice sample, and 871 were unique to the *Xoc* GX01 T3SD infiltrated rice sample. (Fig 4A) Interestingly, there were 442 DEGs between both samples, with 181 of them upregulated with *Xoc* GX01 T3SD infiltration, while other 261 are downregulated (Fig 4B; S2 Table). A total of 328 DEGs identified had functional annotations, whereas 114 DEGs have unknown functions (S2 Table). We analysed the functions of all DEGs using gene ontology (GO) enrichment. A total of 30 significant GO terms were identified from the rice DEGs. Most of the enriched terms involved anchoring to the membrane or were related to the cell wall, cell growth and redox reactions (Fig 4C). Kyoto Encyclopedia of Genes and Genomics (KEGG) pathway enrichment analysis showed that the most changed pathways were biosynthesis of secondary metabolites, phenylalanine metabolism, phenylpropanoid biosynthesis, and flavonoid biosynthesis (Fig 4D; Table 4).

**Table 4:** Rice Differential Group (RDG) data DEGs enrichment on rice (dosa) pathways.

Term	ID	DEGs	Background	P-Value
Phenylpropanoid biosynthesis	osa00940	20	125	3.73E-05
Phenylalanine metabolism	osa00360	16	99	2.33E-04
Biosynthesis of secondary metabolites	osa01110	56	779	3.69E-04
Flavonoid biosynthesis	osa00941	5	19	3.26E-02

Note: Only significant enriched pathways (P < 0.05) were listed. DEGs means the differentially expressed gene counts in designated pathway. Background mean the total gene count in designated pathway.

Based on gene function annotation, we identified that 70 DEGs are related to plant defence (S2 Table). Upon wild-type *Xoc* GX01 infection, the changed defense-related genes included two cell wall-associated receptor kinases (WAKs), OsWAK82 and OsWAK112, two NBS-LRR type R-genes (OsRPM1 and OsRPS2), and two Receptor-like cytoplasmic kinase (OsRLCK153 and OsRLCK298). These genes have been previously shown to be directly activated by ligand-activated pattern recognition receptors (PRRs) and take part in recognition of conserved pathogen-associated molecular patterns (PAMP) by initiating pattern-triggered immunity (PTI) [12,13]. We also identified that the expression of OsMEK3 (Os04t0339800-00) was not expressed in response to *Xoc* infection. OsMEK3 has been shown to be important in the mitogen-activated protein kinase signalling cascade. Furthermore, a number of defence-related transcription factors were also shown to be suppressed, including five WRKY transcription factors[14] and four NAC (NAM/ATAF/CUC) transcription factors[15,16]. Other defence-related genes that appear to be altered in response to *Xoc* infection included those involved in callose deposition and phytoalexin accumulation (S2 Table). This set of genes included six phenylalanine ammonia-lyases, two chalcone isomerases and two genes involved in momilactone biosynthesis.

We confirmed that the wild-type *Xoc* strain GX01 encoded 20 transcription activator-like effectors (TALEs) identical or similar to the model *Xoc* strain BLS256 (S3 Table). Given that TALEs are well documented to activate host gene transcription we examined all potential TALE target genes in rice. In our transcriptome data from rice infected with either wild-type *Xoc* GX01 or *Xoc* GX01 T3SD strain, 12 TALE target genes showed similar expression patterns in a previous research (Table 5) [17]. Importantly, a well-known rice susceptibility gene OsSultr3;6, which is targeted by *Xoc* TALE BLS256_tal2g is one of the 12 TAL effector targets confirmed.

**Table 5:** Differential expression of TALE target gene in *Xoc* infected rice leaves.

TAL effector	Equivalent TALE in BLS256	Target ID	T3SD FPKM	WT FPKM	Fold change	q-Value	Fold Change Mock-Xoc 96 h	q (Mock-Xoc)
GX01_Tal7	BLS256_Tal6	Os01g0496900	6.09	24.84	-2.03	4.87E-03	1.53	8.00E-02
GX01_Tal7	BLS256_Tal6	Os09g0466100	0.27	2.87	-3.41	4.87E-03	1.97	3.60E-02
GX01_Tal7	BLS256_Tal6	Os12g0624900	7.1	30.2	-2.09	4.87E-03	1.65	2.20E-04
GX01_Tal8a	BLS256_Tal5a	Os02g0251900	0.59	36.56	-5.95	4.87E-03	4.93	4.10E-07
GX01_Tal9a	BLS256_Tal4a	Os03g0575200	28.6	225.37	-2.98	4.87E-03	1.96	2.20E-04
GX01_Tal9b	BLS256_Tal4b	Os09g0494600	0.64	5.44	-3.08	4.87E-03	2.45	8.00E-03
GX01_Tal9c	BLS256_Tal4c	Os06g0567200	3.24	63.16	-4.29	4.87E-03	7.15	2.70E-10
GX01_Tal10b	BLS256_Tal3b	Os02g0555300	0.35	6.72	-4.27	7.84E-02	5.31	4.00E-07
GX01_Tal10b	BLS256_Tal3b	Os05g0342100	0.27	25	-6.55	1.16E-01	4.42	3.40E-08
GX01_Tal10b	BLS256_Tal3b	Os07g0549100	0	3.68	-inf	4.87E-03	2.4	2.60E-02
GX01_Tal10c	BLS256_Tal3c	Os02g0705500	1.83	33.11	-4.18	1.00E+00	2.24	1.90E-03
GX01_Tal10c	BLS256_Tal3c	Os03g0171700	0.25	171.28	-9.42	1.33E-01	3.84	3.60E-02
GX01_Tal11b	BLS256_Tal2c	Os03g0122300	7.77	132.25	-4.09	4.87E-03	3.11	1.10E-02
GX01_Tal11c	BLS256_Tal2d	Os04g0581000	1.46	248.65	-7.41	4.87E-03	10.49	3.90E-07
GX01_Tal11g	BLS256_Tal2g	Os01g0719300	0.65	229.29	-8.45	4.87E-03	9.59	1.30E-06
GX01_Tal11g	BLS256_Tal2g	Os06g0678800	0.94	60.93	-6.02	4.87E-03	6.88	4.30E-08

Note: Fold change shows log2(T3SD_FPKM/WT_FPKM), represents transcript abundance in leaves inoculated with *Xoc* GX01 NK4122(ΔhrcC, type three secretion system defect) relative to *Xoc* GX01 wild type “Fold Change Mock-Xoc 96 h” data are from Cernadas et al., 2014, which represented fold change in transcript abundance at 96 h in *Xoc*-inoculated leaves relative to mock-inoculated leaves [17]. q-value < 5.00E-2 and |fold change| > 1 are the threshold for significant gene expression charge. OsSultr3;6 was referred as Os01g0719300 in this table.

Several rice genes that have not previously been shown to be responsive to *Xoc* infection were also identified. Several genes involved in cell elongation reactions were induced over 10-fold including alpha-expansin, beta-expansin, and xyloglucan endotransglucosylases (S2 Table). The overexpression of genes encoding these enzymes has been shown reducing the cell wall strength in *Arabidopsis*, and increasing susceptibility to other plant pathogens like *Botrytis cinereal* and *Candidatus Liberibacter asiacitus* [18]. We suspect these genes are exploited by *Xoc* to work as cell wall loosening factors that contribute to rice susceptibility.

Taken together, the data reveal that *Xoc* infection has a significant impact on the rice transcriptome and that a proportion can be attributed to the presence of a functional T3SS. The data also reveal a new set of host genes that were thought not to be altered during *Xoc* infection but might to be direct or indirect targets of the T3SS (and T3SE), suggesting valuable targets for further study.

Although we tried to avoid the limited number of reads isolated from rice samples infected with *Xoc* GX01 T3SD strain by increasing the depth of sequencing, it still yielded lower numbers. Despite this we carried out standard analysis with this caveat in mind (Table 6, S4 and S5 Tables). There were 4773 transcripts present in *Xoc* GX01 wild type and T3SD strain total *in planta*, including 4033 annotated genes, 340 novel genes and 400 small RNAs. Among these annotated gene transcripts, 3910 transcripts were identified in both strains, with 36 transcripts uniquely identified in wild type GX01, and 87 uniquely identified in T3SD mutant. There were 175 DEGs between two strains, and 62 of them show a higher expression in T3SD mutant, while other 113 were lower. The *Xoc* GX01 T3SD DEG profile shows a lot of the genes that were involved in virulence and regulated in the wild-type during plant infection were not affected in this mutant, suggesting the importance of the T3SS to in planta infection and propagation. Interestingly, GO enrichment showed there was still an overall downregulation of genes involved in encoding proteins for ribosomal assembly, transcription complex assembly, polysaccharide metabolism activity and ATP biosynthesis and the upregulation of genes involved in antioxidant and detoxification processes (S4 and S5 Tables). These observations are consistent with a recent study of a T3SE free mutant of *Pseudomonas syringae* pv. *Tomato* DC3000 (*Pto*) which was profiled infecting the model plant *Arabidopsis thaliana* [19]. Conversely, the *Xoc* T3SD strain did not show any expression changes in genes involved in flagella mobility but did appear to show the up regulation of genes involved in stress response process (RpoH, XOC_0605 and XOC_3651) which is very different to the *Pto* T3SE free mutant *in planta* expression profile and also the *Xoc* wild-type infecting rice.

**Table 6:** *in planta* DEGs data between T3SDXOC and WTXOC enreichment on *Xoc* (xor) pathways.

Pathway	ID	DEGs	Background	P-Value
Ribosome	xor03010	25↓	59	5.52E-09
Aminobenzoate degradation	xor00627	3↑	12	1.89E-02
Fatty acid degradation	xor00071	3↑	12	1.89E-02

Note: Up arrow means upregulated in T3SDXOC, down arrow means downregulated in T3SDXOC. Only significantly enriched pathways (P < 0.05) were listed. DEGs mean the differentially expressed gene counts in designated pathway. Background mean the total gene count in designated pathway.

### Analysis of *Xoc* differential expression genes between *in planta* and in media environment

**Fig 5:**
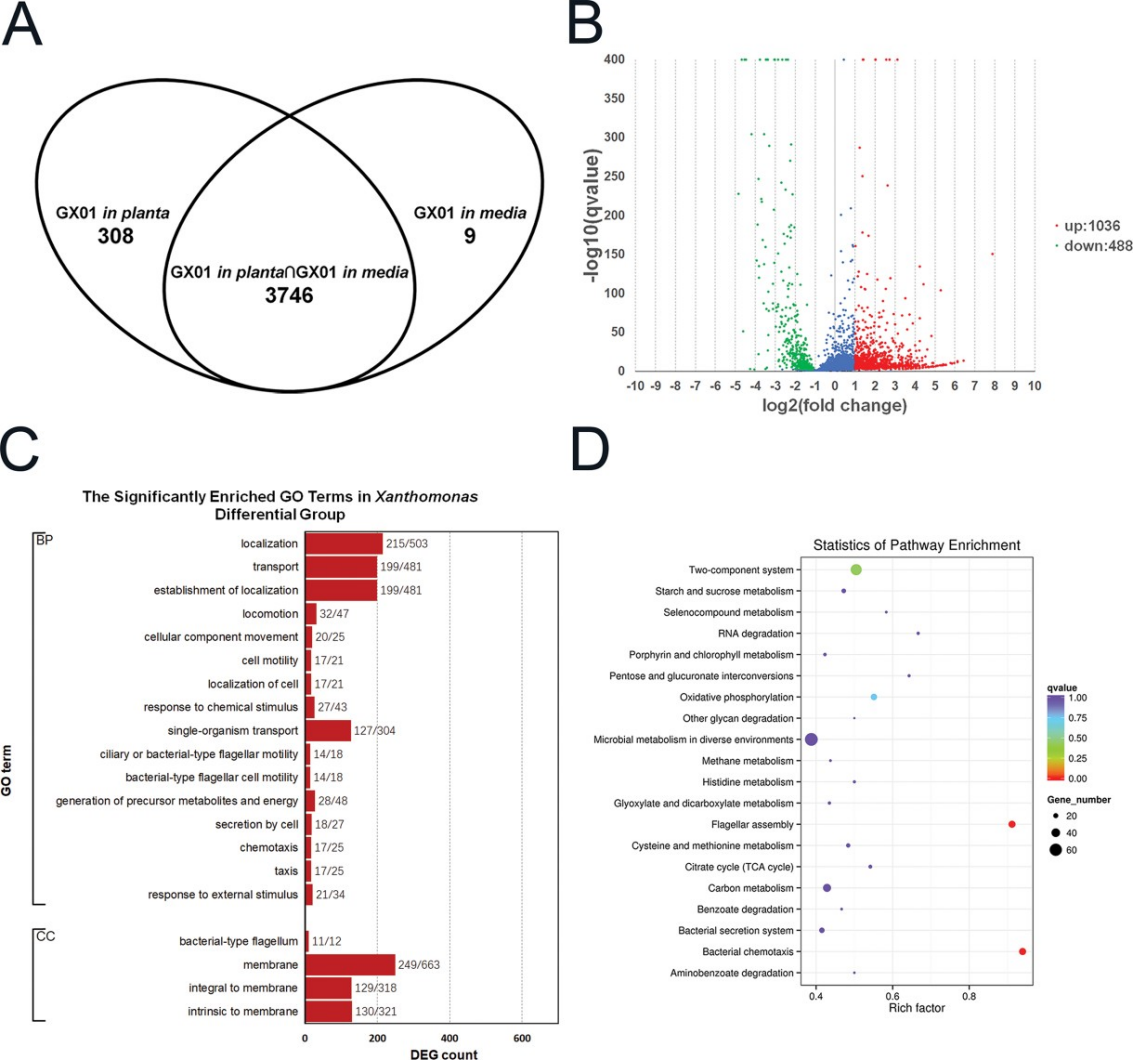
Differential gene expression (DEG) of *Xanthomonas oryzae* pv. *oryzicola* in culture and during plant infection. **(A)** A Venn diagram showing DEGs between GX01 grown *in planta* and in media. The data represent the numbers of genes with FPKM > 1. **(B)** A volcano plot showing DEGs between GX01 grown *in planta* and in media. A log2(fold change) > 1 and q-value < 0.005 were used as the cutoff values. **(C)** Histogram showing GO enrichment analysis of DEGs in the *Xanthomonas* Differential Group (XDG). The y-axis indicates the GO terms, and the x-axis indicates the numbers of DEGs shown as DEG number/background number. All terms shown have corrected P-values < 0.05. BP: biological process; CC: cellular component; **(D)** Bubble chart showing KEGG enrichment analysis of DEGs in the *Xanthomonas* Differential Group (XDG). The y-axis indicates the enriched pathways, and the x-axis indicates the rich factor. Bubble color indicates q-value and the size indicates the DEG count. The chart showed most enriched 20 pathways.

To investigate how *Xoc* adapts to the *in planta* environment, we compare the transcriptome in planta with that in complex media. A series of gene expression changes occurred in *Xoc* in response to rice infection and growth in complex media. Our analysis identified 4221 transcripts present in *Xoc* from growth *in planta* and in complex media, including 4063 annotated genes, 106 novel genes and 52 small RNAs. Only take annotated gene transcripts in count, 3746 transcripts were identified in both growth conditions, with 308 transcripts uniquely identified *in planta*, and 9 uniquely identified in complex media (Fig 5A). A total of 1524 genes, covering almost one-third of the *Xoc* annotated genes, were significant changed, of which, 1036 were upregulated and 488 downregulated *in planta* (Fig 5B; S6 Table). Using the same GO enrichment analysis method used initially for our rice data, we identified a total of 20 significant GO terms from *Xoc* GX01 DEGs between growth in *in planta* and media (Fig 5C). The individual DEGs were spread across a wide range of biological processes that involved localization, locomotion, chemotaxis, metabolism, and secretion according to their annotations. Another large proportion of DEGs fell into the cellular component category, including membrane, bacterial-type flagellum, integral to membrane, and intrinsic to membrane. Strikingly, many of the alterations in genes associated with membrane structural changes were only found in *Xoc* grown *in planta*. Furthermore, the KEGG pathway analysis, found that the genes downregulated *in planta* were enriched in flagellar assembly and bacterial chemotaxis pathways, whereas the genes upregulated *in planta* were enriched in the bacterial secretion system pathway (Fig 5D; Table 7). These findings suggest that a wide range of gene expression changes occurred specifically in *Xoc* during adaptation to the plant environment.

**Table 7:** *Xanthomonas* Differential Group (XDG) DEGs data enrichment on *Xoc* (*xor*) pathways.

Pathway	ID	DEGs	Background	P-Value
Flagellar assembly	xor02040	31↓	34	1.19E-05
Bacterial chemotaxis	xor02030	30↓	33	1.19E-05
Bacterial secretion system	xor03070	16↑	53	3.34E-02

Note: Up arrow means upregulated *in planta*, down arrow means downregulated *in planta*. Only significant enriched pathways (P < 0.05) were listed. DEGs means the differentially expressed gene counts in designated pathway. Background means the total gene count in designated pathway.

We then examined the gene expression changes and gained a better understanding of the potential mechanisms that *Xoc* deployed to sense and response to the environment in particular living *in planta*. We identified changes in well-known sensory systems including two-component signal systems (TCS) and transcription regulators that serve as basic stimulus-response systems to sense and respond to various changes in environmental conditions [4,20]. 42 TCS genes were significantly changed in response to growth *in planta* and in media (S6 Table). This included 16 sensors, 18 response regulators, and 8 sensor-response regulator hybrids. When investigating their functions, we found that a remarkable downregulation *in planta* occurred of the genes encoding CheA-CheYBV and RegS-RegR, showing a potential reduction in chemotaxis[4] and antioxidant[21] activity by *Xoc*. We also saw the upregulation of genes encoding TctE-TctD and RaxHR/RaxX which are known to be involved in tricarboxylic acid transport regulation[22] and virulence factor activity[23], respectively.

Except for TCS, transcription regulator proteins (TRPs) play a major role in controlling bacterial gene transcription in response to environmental changes [20]. Our analysis revealed a total of 38 TRPs that were significantly changed under the conditions of growth (S6 Table). Several of these TRPs were known to control carbon metabolism. These included 4 transcriptional activators, *pobR*, *pcaQ*, *padR* and *vanR* that were upregulated exclusively *in planta*, indicating the need for catabolism for benzoic and phenolic acids during infection[24]. And the benzoate degradation genes were upregulated as an outcome of this. (S6 Table) Additionally, the observed upregulation of *malT* and *cdaR* suggest an increased demand on maltose uptake and utilization *in planta*. Moreover, our results suggested the demand for amino acid uptake and biosynthesis given that the gene encoding leucine-responsive regulatory protein Lrp had a higher expression while the proline utilization regulon repressor *putA* was down regulated *in planta*.

It is clear that *Xoc* initiated various responses to the environmental changes and as highlighted above this included genes involved in cell motility, flagellar assembly and flagellar-derived movement (S6 Table). More specifically, genes that involved in virulence factor synthesis, disease and growth were highly regulated *in planta*. As expected, the *hrp* gene cluster, which encodes components of the T3SS mechanism, was upregulated together with several T3SEs, several type two secretion effectors and one set of type six secretion systems (S6 Table). Furthermore, small molecule transmembrane transporter essential for virulence factor export and nutrition uptake was also strongly regulated. A total of 88 type one transporters and other permease genes were differentially expressed in *Xoc in planta*, with 69 of these genes upregulated and 19 genes downregulated. (S6 Table). The majority of these genes encoded proteins involved in nutrient uptake including carbohydrate, amino acid and metal ion transporters (S6 Table). This is likely a result of *Xoc* savaging for nutrients *in planta*. A broad range of downregulation in carbohydrate metabolism also occurred *in planta*, involved in glycolysis, citrate cycle, pentose phosphate pathway and some other pathways. Together with the downregulation in oxidative phosphorylation, *Xoc* shows a major reduction in energy consumption *in planta*.

## Conclusions

In summary, here we developed and employed a dual RNA-seq approach to examine a real-world bacterial plant disease. Combining our *Xoc* rice plant infection model with a robust next-generation sequencing approach we have developed a reliable real-time *in situ* transcriptome approach for the simultaneous monitoring of rice and *Xoc* gene expression levels during infection. Compared to earlier studies that used a conventional microarray to examine bacterial grown in laboratory induction medium that mimicked an *in planta* environment [25], our method was more reliable and resulted in better bacterial transcript coverage and sensitivity with up to 500-fold more DEGs, but also provided transcript coverage of the host plant, which benefited from both a probe-free process and a real *in planta* environment [26]. Furthermore, compare to previous *in planta* pathogenic bacteria RNA-sequencing methods that involve bacteria isolated and enriched [19], using deep sequencing can provide transcript coverage of the host plant but also avoid the potential processing issues that alter the transcriptome during the bacterial isolation[27].

Overall, our results indicated that *Xoc* considerably altered rice transcriptome during infection, directly or indirectly dependent on T3SS. Rice genes appeared to be targeted by bacterial pathogens, such as ones involved in callose deposition and phytoalexin accumulation. We also observed several gene expression changes that not previously known related to T3SEs, e.g. genes for auxin-induced cell elongation. Meanwhile, the T3SD strain transcriptome *in planta* was characterized by downregulation of ATP, protein and polysaccharide synthesis, and upregulation of antioxidation and detoxification related genes, revealing that T3SD strain faced serious starvation and oxidation stresses *in planta* without a functional T3SS. Furthermore, it appears that *Xoc* specifically altered its own global gene expression profile for survival *in planta*, with alterations to genes involved in transmembrane transports, reduction of chemotaxis, reduced activation of flagellar locomotion, and reduction in energy consumption. Of course, further work is warranted to understand the functionality and impact on disease that these gene expression changes have on both in *Xoc* and rice during infection. However, comparing these findings to a recent RNA-seq study examining *Pto* gene expression infecting the model plant *A*. *thaliana* it is clear to see that *Xoc* uses very different functions to adapt to rice infection. This highlights the importance of examining plant pathogenic bacteria infecting its true host in order to gain meaningful insight. We feel that with minor adjustment this approach can be used to obtain dual RNA-seq data from many other bacterial-plant interactions.

## Materials and methods

### Bacterial strains and rice cultivar used in this study

*Xanthomonas oryzae* pv. *oryzicola* (*Xoc*) strain GX01 is a rifampicin-resistance spontaneous mutant of strain LT4 (designated *Xoc* GX01 in this study), which was isolated from the rice leaf with typical BLS symptoms in Liantang Town of Hezhou City of Guangxi, in the central area of South China rice growing regions [28]. *Xoc* strain NK4122 is a T3SS-defective mutant carrying a *hrcC* insertional mutant in the wild-type *Xoc* GX01 background (designated *Xoc* GX01 T3SD in this study). *Oryza sativa* L. cv. Nipponbare was used in this study as a susceptible host of *Xoc* GX01.

### Culture and growth conditions

*Xoc* strains were grown in rich medium named nutrient broth (NB) (1% polypeptone, 0.5% yeast extract, 1% sucrose, pH 6.8) or on nutrient agar (NA) (NB with 1.5% agar) plates at 28°C [29]. Antibacterial grow selection for the GX01 strain was 50 μg/ml rifampicin but for the NK4122 strain an additional 25 μg/ml kanamycin was added. To prepare *Xoc* bacterial cultures for RNA-seq, *Xoc* strains were grown for 24 h at 28°C in NB medium, and the cells were collected by centrifugation and re-suspended in sterilized water. *Xoc* suspension was adjusted to Optical Density (O.D.)_600_ = 1 (approximately 1.0 × 10^8^ CFU/mL), 100 μL of the *Xoc* suspension was added into 10 mL fresh NB medium and grown for 24 h at 28°C. These cells were collected by centrifugation and the medium was discarded. Cell pellets were snap frozen in liquid nitrogen, then stored at −80°C immediately.

### Rice infection model

Rice (*Oryza sativa* L. cv. Nipponbare) seeds were germinated by submersion in water, followed by incubation in the dark for two days at 37°C. Five germinated seeds were planted in a small pot containing autoclaved soil. The plants were grown in the greenhouse under a 14/10-h light/dark cycle with a temperature controlled between 28°C and 30°C till the fourth leaf begins sprouting (about 14 days). Before inoculated, *Xoc* strains were grown for 24 h at 28°C in NB medium, and the cells were collected by centrifugation and re-suspended in sterilized water. The concentration of the *Xoc* suspension was adjusted to O.D._600_ = 0.5 (approximately 5.0 × 10^7^ CFU/mL) prior to infiltration.

Two-week-old rice seedlings were inoculated with the *Xoc* suspension using a syringe infiltration method [30]. For each plant, the second and third leaves were picked, and 2/3 of the leaf surface was infiltrated. The infiltrated leaf part was collected 24 h after inoculation, snap frozen in liquid nitrogen, and stored at −80°C immediately.

For virulence test, tillering stage rice (about 4 to 6 weeks old) was inoculated with the *Xoc* suspension using the same method above. For each plant, the second and third leaves were picked, but for each leaf, only two infiltrations on both sides of midrib were made at 1/3 from leaf tip. And the water soaking lesion was measured at 14 days after infiltration.

### Sequencing approach

To analyse the rice response to *Xoc* infection, the *Xoc* GX01 strain (wild type) was compared to the *Xoc* GX01 T3SD, a T3SS-defective mutant (a *hrcC* insertional mutant named NK4122) during infection of rice leaves using dual RNA-seq. Deeper sequencing than usual (> 20 GB per mixed sample) was employed to overcome the RNA quantity difference between the rice and *Xoc*. Additionally, an rRNA removal strategy was used which is typical for long non-coding RNA (lncRNA) sequencing instead of poly-T enrichment during library construction. The mRNAs from the plants and bacteria were reverse transcribed simultaneously. In conjunction with the dual RNA-seq experiments, *Xoc* gene expression was profiled during growth in complex media. For this we employed a standard RNA-seq approach to examine GX01 grown in NB medium [31].

### Isolation of total RNA from infected plant cells (or bacterial culture)

Total RNA was extracted from infected plant samples or bacterial culture using mirVana Isolation Kit (Ambion Life Technologies) according to the manufactures instructions. After extraction the RNA integrity was assessed using the RNA Nano 6000 Assay Kit for the Bioanalyzer 2100 system. A total of 3 μg of RNA per sample was used as the input material for the RNA sample preparations.

### cDNA library construction and sequencing

Before cDNA library preparation, ribosomal RNA was removed using the Epicentre Ribo-zero rRNA Removal Kit (Epicentre, USA), and the rRNA-free residue was cleaned by ethanol precipitation. Subsequently, sequencing libraries were generated using the rRNA-depleted RNA with the NEBNext Ultra Directional RNA Library Prep Kit for Illumina (NEB, USA) following the manufacturer’s recommendations. rRNA-depleted RNA was fragmented by mixed with NEB Next First Strand Synthesis Reaction Buffer and incubated at 94°C for 15 minutes. First-strand cDNA was synthesized using random hexamer primers and the M-MuLV Reverse Transcriptase (RNase H^-^). Second-strand cDNA synthesis was performed subsequently using DNA Polymerase I and RNase H. In the reaction buffer dNTPs, dTTP was replaced with dUTP. The remaining overhangs were converted into blunt ends via exonuclease/polymerase activity. After adenylation of the DNA fragment 3’ ends, the NEBNext Adaptor with a hairpin loop structure was ligated to prepare for hybridization.

To preferentially select cDNA fragments 150–200 bp in length, the library fragments were purified with the AM Pure XP system (Beckman Coulter, Beverly, MA, USA). After that, 3 μL of the USER Enzyme (NEB, USA) was incubated with size-selected, adaptor-ligated cDNA at 37°C for 15 min, followed by incubation for 5 min at 95°C prior to the PCR. The PCR was performed with the Phusion High-Fidelity DNA polymerase, Universal PCR primers and Index (X) Primer.

Finally, the products were purified (AM Pure XP system), and the library quality was assessed on the Agilent Bioanalyzer 2100 system.

### Clustering, sequencing, read mapping and data analysis

Clustering of the index-coded samples was performed on a cBot Cluster Generation System using the TruSeq PE Cluster Kit v3-cBot-HS (Illumina) according to the manufacturer’s instructions.

After cluster generation, the libraries were sequenced on the Illumina Hiseq 2000 platform, and 125-bp paired-end reads were generated. After removing reads containing the adapter, reads containing poly-N, and low-quality reads, clean reads were obtained. The Q20, Q30 and GC contents of the clean reads were calculated. All downstream analyses were based on the clean reads.

Reference genome and gene model annotation files were downloaded directly from the genome NCBI ftp server. For rice transcript mapping, we used the *Oryza sativa* Japonica Group (Assembly ID = 22512) as a reference. Fo*r Xanthomonas oryzae* pv. *oryzicola* strain GX01 transcript mapping, we used *Xoc* BLS256 (Assembly ID = 357911) as a reference. An index of the reference genome was built using Bowtie v2.0.6, and paired-end clean reads were aligned to the reference genome using TopHat v2.0.9.

### Quantification and differential gene expression analysis and gene enrichment analyses

Cuffdiff (v2.1.1) was used to calculate the FPKMs of the genes in each sample [32]. Gene FPKMs were computed by summing the FPKMs of transcripts in each gene group. The FPKMs were calculated based on the lengths of the fragments and the read counts mapped to these fragments. Cuffdiff provides statistical routines for determining differential expression in digital transcript or gene expression data using a model based on the negative binomial distribution [32]. For biological replicates, transcripts or genes with Q-values < 0.05 and an absolute value of log2(fold change) > 1 were assigned as differentially expressed. For non-biological replicates, P-adjust < 0.005 and an absolute value of log2(fold change) > 1 were set as the thresholds for significantly differential expression.

Gene ontology (GO) enrichment analysis of the differentially expressed genes was implemented using the GOseq R package, in which gene length bias was corrected. GO terms with a corrected q-value < 0.05 were considered significantly enriched for the DEGs.

We used the KOBAS software to test the statistical enrichment of the DEGs in Kyoto Encyclopedia of Genes and Genomics (KEGG) pathways. We use Fisher's exact test follow by Benjamini and Hochberg procedure to determine the false discovery rate (q-value). Pathways with a q-value < 0.05 were considered significantly enriched for the DEGs.

### Quantitative reverse transcription polymerase chain reaction (qRT-PCR)

For qRT-PCR analysis, total RNA was reverse transcribed into cDNA using RevertAid First Strand cDNA Synthesis Kit (Thermo Scientific). Using FastStart Universal SYBR Green Master (Roche) on qTOWER 2.0 Real-Time Thermocycler (Analytik Jena), qRT-PCR experiments were performed in triplicates.

Gene-specified primers were designed for selected genes using Primer-BLAST [33]. 2^–ΔΔCT^ method was used for calculating the relative target gene expressions [31].The primers that were used to target specific genes in rice and bacteria are listed on S1 Table.

## Supporting information

S1 Table Verification of DEGs Using qRT-PCR. (XLSX)

S2 Table Differentially expressed genes within the rice differential group. (XLSX)

S3 Table TALEs RVDs in *Xoc* GX01. (XLSX)

S4 Table Differentially expressed genes between WT and T3SD *Xoc* strains *in planta*. (XLSX)

S5 Table *in planta* DEGs data between T3SDXOC and WTXOC for DEG GO enrichment. (XLSX)

S6 Table Differentially expressed genes within the *Xanthomonas* differential group. (XLSX)
